# Broiler Farming in Africa vs. the EU: Divergent Systems, Shared Challenges, and Future Directions—A Review

**DOI:** 10.3390/ani16152399

**Published:** 2026-08-03

**Authors:** Agnieszka Ludwiczak, Adaramoti Temitope

**Affiliations:** Department of Animal Breeding and Product Quality Assessment, Poznan University of Life Sciences, Szydlowska 50, 60-656 Poznan, Poland; temitopeadaramoti@gmail.com

**Keywords:** broiler production, Europe, Africa, chicken husbandry

## Abstract

Background: Broiler production systems in Africa and the European Union (EU) differ substantially in terms of housing design, environmental control, welfare outcomes, and regulatory oversight. Understanding these differences is essential for identifying region-appropriate strategies for sustainable poultry production. Objectives: This review aimed to compare broiler housing systems, welfare outcomes, environmental management, and regulatory frameworks in Africa and the EU. Methods: This review followed some aspects of the PRISMA 2020 guidelines. Searches were conducted in Scopus, Web of Science, PubMed, and CAB Abstracts from January 2023 to January 2025. The grey literature from the FAO, EU institutions, and NGOs was also screened. Eligible studies examined broiler housing systems, welfare indicators, environmental control, or regulatory frameworks in African or EU contexts. Two reviewers independently screened records, extracted data, and assessed risk of bias using the SYRCLE tool. No meta-analysis was performed due to heterogeneity; results were synthesized narratively. Results: The search identified 86 records; 83 remained after duplicate removal. After screening, 45 studies met the inclusion criteria. EU systems were characterized by climate-controlled indoor housing, strict welfare regulations, and integrated supply chains. African systems ranged from intensive open-sided houses to semi-intensive and backyard systems, with major constraints including heat stress, limited access to veterinary care, and high feed costs. These systems also differ markedly across African regions, reflecting variation in infrastructure, market integration, and climatic conditions. Risk of bias was moderate across most studies. The certainty of the evidence was low to moderate due to heterogeneity in study designs and reporting. Limitations: Heterogeneous study designs prevented quantitative synthesis. Reporting quality varied, and few African studies provided detailed welfare metrics. Conclusions: Broiler production systems in Africa and the EU reflect distinct climatic, economic, and institutional contexts. Sustainable improvements require region-specific strategies rather than universal housing models. In this review, region-specific strategies refer to climate-adaptive housing designs, modular environmental control technologies, and management approaches aligned with local infrastructure, market structures, and resource availability.

## Simple Summary

Broiler production systems in Africa and the European Union differ significantly due to variations in climate, economic resources, infrastructure, and regulatory frameworks. In the EU, broiler farming is highly intensive, technologically advanced, and guided by strict animal welfare and environmental standards. In contrast, African systems are more diverse, ranging from commercial operations to small-scale and backyard farming adapted to local conditions. African production systems also vary substantially across North, West, East, and Southern Africa, each shaped by distinct climatic pressures, market structures, and infrastructural constraints. Despite these differences, both regions face similar challenges in balancing productivity, animal welfare, and sustainability. This review highlights key differences in housing, management, and market structures, while identifying opportunities to improve broiler production through context-specific solutions and knowledge exchange across regions.

## 1. Introduction

This review was conducted and reported in accordance with the PRISMA 2020 guidelines to ensure methodological transparency and reproducibility. Broiler production has become one of the central pillars of the global livestock sector, contributing significantly to food security, economic development, and the supply of dietary protein [1]. Advances in genetics, nutrition, and housing technologies have transformed chicken meat from a relatively scarce commodity into one of the most accessible animal proteins worldwide [2,3]. However, these rapid gains have also introduced new welfare and sustainability concerns. Modern broilers, selected for extremely fast growth, face physiological challenges including leg disorders, metabolic strain, and reduced mobility [4,5]. These biological constraints make housing design and environmental management increasingly important determinants of welfare outcomes. Housing conditions play a critical role in shaping broiler welfare. Dawkins et al. demonstrated that environmental quality, ventilation, temperature, litter condition, and lighting have a stronger influence on welfare than stocking density alone [6]. This finding underscores the need to evaluate housing systems holistically rather than focusing solely on space allowance. The divergence in broiler production systems across regions reflects differences in climate, infrastructure, governance, and consumer expectations. In the EU, broiler production is embedded within highly integrated supply chains governed by strict animal-welfare, environmental, and food-safety regulations [7]. These systems reflect growing societal concerns around sustainability and ethical livestock production [8]. In contrast, African broiler production operates across a spectrum of systems ranging from modern intensive farms to traditional backyard setups, shaped by infrastructural limitations, socio-economic realities, and cultural practices [1,9]. African systems also differ substantially across North, West, East, and Southern Africa, each characterized by distinct climatic pressures, levels of market integration, and infrastructural capacity. Despite these structural differences, both regions face mounting pressure to balance efficiency, animal welfare, and sustainability within evolving market environments [10]. This review therefore compares broiler housing systems, regulatory frameworks, and market drivers across Africa and the EU, highlighting how regional realities shape production strategies and identifying opportunities for cross-regional learning.

Despite extensive research on broiler production systems, existing studies predominantly focus on isolated dimensions such as animal welfare, productivity, or environmental impacts within specific regions. Comparative analyses across regions, particularly between the European Union and African contexts, remain limited and are often conducted without a consistent analytical framework. Moreover, differences in housing systems, regulatory environments, and socio-economic drivers are rarely examined in an integrated manner. As a result, there is a lack of structured, cross-regional assessments that enable meaningful comparison and synthesis of broiler production systems under contrasting conditions.

To address this gap, the present study adopts a comparative analytical framework that evaluates not only how systems differ but also why these differences emerge and under which conditions specific housing or management strategies are feasible, infeasible, or only partially transferable across regions. To guide this analysis, the study is structured around the following research questions: RQ1. How do environmental control strategies differ between broiler housing systems in Africa and the EU? RQ2. How do these environmental control strategies influence key welfare and production outcomes under temperate versus tropical climatic conditions?

## 2. Materials and Methods

### 2.1. Protocol and Registration

This review followed some aspects of the PRISMA 2020 guidelines. No protocol was registered, and no prior protocol document was prepared. References were managed using Zotero (v7.0.15; Corporation for Digital Scholarship, Vienna, VA, USA).

### 2.2. Eligibility Criteria

Studies were eligible if they met all of the following criteria:

- Population: Broiler chickens (*Gallus gallus* domesticus).
- Concept: Housing systems, environmental management, welfare outcomes, or regulatory frameworks.
- Context: African or European Union production systems.
- Study type: Empirical studies (experimental or observational), reviews, or official reports with extractable data.
- Language: English.
- Publication years: No restrictions.

Exclusion criteria:

- Studies not focused on broiler housing or welfare.
- Studies lacking extractable data.
- Conference abstracts without full text.
- Non-English publications.
- Studies on layer hens or indigenous scavenging systems without broiler-specific data.

Studies were grouped into three domains:

- Housing system characteristics
- Welfare and health outcomes
- Regulatory and socio-economic frameworks

### 2.3. Information Sources

Searches were conducted in the following databases:

- Scopus
- Web of Science
- PubMed
- CAB Abstracts

The grey literature sources included FAO reports, EU regulatory documents, and NGO publications. Last search date: 15 January 2025.

### 2.4. Search Strategy and Selection Process

A comprehensive search strategy was developed in consultation with PRISMA 2020 guidelines. Full search strings were constructed using controlled vocabulary and free-text terms, including synonyms for broilers, chickens, poultry, housing systems, welfare, and regional identifiers. No temporal restrictions were applied to the publication years of the included studies; all available years were eligible. The final search was conducted on 15 January 2025.

Scopus (TITLE-ABS-KEY):

TITLE-ABS-KEY (broiler* OR “meat chicken*” OR chicken* OR poultry*)

AND (“housing system*” OR “production system*” OR “environmental control” OR ventilation OR “stocking density” OR welfare)

AND (Africa OR “Sub-Saharan Africa” OR “European Union” OR Europe OR EU)

Web of Science (TS = Topic):

TS = (broiler* OR “meat chicken*” OR chicken* OR poultry*)

AND (“housing system*” OR “production system*” OR “environmental control” OR ventilation OR “stocking density” OR welfare)

AND (Africa OR “Sub-Saharan Africa” OR “European Union” OR Europe OR EU)

PubMed (Title/Abstract):

(broiler* [Title/Abstract] OR chicken* [Title/Abstract] OR poultry [Title/Abstract])

AND (“housing system” [Title/Abstract] OR “production system” [Title/Abstract]

OR “environmental control” [Title/Abstract] OR ventilation [Title/Abstract]

OR “stocking density” [Title/Abstract] OR welfare [Title/Abstract])

AND (Africa [Title/Abstract] OR Europe [Title/Abstract] OR “European Union” [Title/Abstract])

CAB Abstracts (All fields):

(broiler* OR chicken* OR poultry*)

AND (“housing system*” OR “production system*” OR “environmental control”

OR ventilation OR “stocking density” OR welfare)

AND (Africa OR Europe OR “European Union”)

## 3. Results

**Figure 1:**
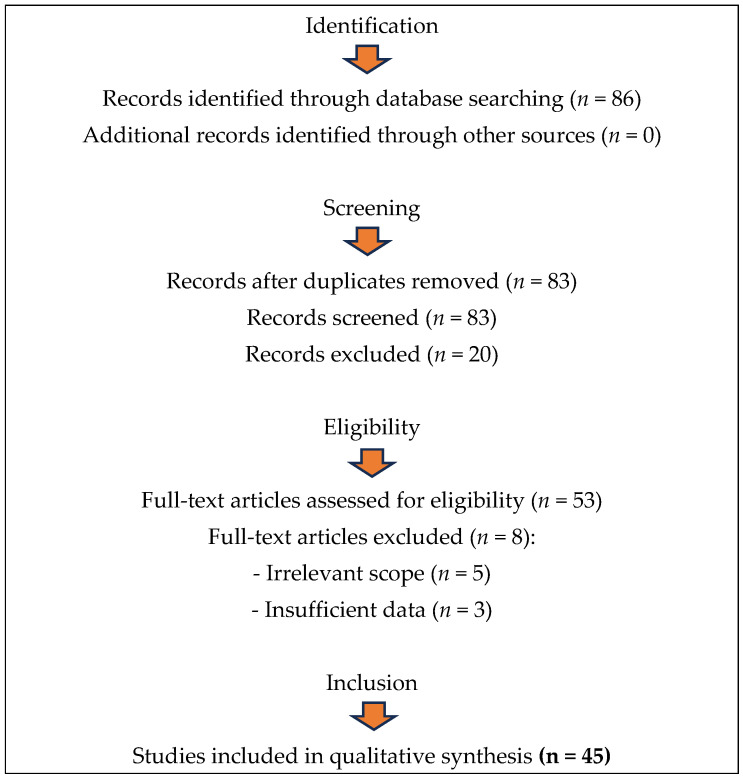
PRISMA flow diagram.

Following the removal of duplicate entries, 83 records remained for screening (Figure 1). Titles and abstracts were evaluated against predefined inclusion criteria, requiring studies to (i) focus on broiler chicken housing systems, (ii) address animal welfare and/or regulatory dimensions, and (iii) pertain to either African or European contexts. During this stage, 20 records were excluded for not being relevant to the study’s scope. A total of 53 full-text articles were assessed for eligibility. Studies were required to be either empirical or review-based, include a clear discussion of housing systems and associated welfare outcomes, and incorporate a regulatory or policy perspective. Full-text screening resulted in the exclusion of 8 articles for the following reasons: irrelevant scope (*n*= 5) and insufficient data (*n* = 3). Ultimately, 45 studies met all inclusion criteria and were incorporated into the qualitative synthesis. These studies formed the basis for the comparative analysis of broiler chicken housing systems, animal welfare considerations, and regulatory frameworks in Africa and Europe.

### 3.1. Study Selection

Broiler housing systems in Africa and the European Union differ markedly in their structural design, environmental control, welfare outcomes, and regulatory oversight. These differences reflect the broader biophysical, economic, and institutional constraints that shape which housing systems are feasible and sustainable in each region. Table 1 compares broiler housing systems in Africa and the EU, while Table 2a,b outline the dominant constraints across African and European production systems and their implications for production. Together, these tables provide a comparative foundation for understanding how regional realities influence broiler production strategies.

### 3.2. RQ1. How Do Environmental Control Strategies Differ Between Broiler Housing Systems in Africa and the EU?

#### 3.2.1. Environmental Control in EU Systems

Council Directive 2007/43/EC of 28 June 2007, as amended by Regulation (EU) 2017/625, lays down minimum rules for the protection of chickens kept for meat production and applies primarily to intensive indoor floor systems [11,12]. These systems dominate EU broiler production and are characterized by fully enclosed, climate-controlled barns designed to maintain stable environmental conditions throughout the production cycle. A typical climate-controlled broiler house used in the EU is characterised by fully enclosed structure and mechanical ventilation system characteristic of intensive European production. The Directive establishes a baseline maximum stocking density of 33 kg/m^2^, with the option to increase it to 39–42 kg/m^2^ if additional environmental and welfare requirements are met [11]. Despite these regulatory thresholds, scientific evidence suggests that welfare compromises begin at much lower densities. EFSA identifies 11 kg/m^2^ as the threshold above which animal welfare risks increase with high certainty, based on evidence linking stocking density to adverse outcomes, including reduced mobility, increased contact dermatitis, and impaired thermoregulation under suboptimal environmental conditions [13]. This is a persistent gap between legal compliance and optimal welfare. Indoor housing in the EU is also shaped by outcome-based welfare monitoring. Modern welfare assessment frameworks emphasize indicators such as footpad dermatitis, hock burn, gait scoring, and plumage cleanliness [14]. These indicators provide a more accurate reflection of on-farm welfare than input measures alone. Additional studies also confirm that enriched indoor environments improve locomotion, reduce inactivity, and support better leg health in fast-growing broilers, reinforcing the importance of environmental complexity in intensive systems [15,16]. Environmental enrichment is increasingly incorporated into EU systems in response to consumer expectations and welfare science. Studies show that providing perches, pecking substrates, and elevated platforms increases activity levels, reduces stress, and improves leg health [17]. However, even in highly controlled environments, fast-growing broilers continue to experience locomotory disorders and inactivity, reflecting the biological limitations of current genotypes [13]. Air quality remains a central regulatory concern. Annex II of Directive 2007/43/EC specifies that ammonia concentrations must not exceed 20 ppm and carbon dioxide must not exceed 3000 ppm [11]. EFSA recommends even lower ammonia thresholds (≤15 ppm) due to associations with respiratory irritation, ocular damage, and footpad dermatitis [13]. Lighting programs are also regulated. The Directive mandates at least 6 h of darkness, including one uninterrupted 4 h dark period, after the first 7 days of life [11]. EFSA further recommends minimum light intensities of 20 lux, with darker resting areas down to 0.5 lux [13]. Light intensity itself is a key determinant of welfare, with lower intensities associated with reduced activity and poorer leg condition [18].

Across the reviewed EU studies, a consistent pattern emerges: environmental control, particularly ventilation efficiency, ammonia management, and lighting regimes, has a stronger influence on welfare outcomes than stocking density alone. Studies by Dawkins et al. [6], EFSA [13], and related experimental work [15,16] converge on the conclusion that locomotory disorders, footpad dermatitis, and inactivity are primarily driven by environmental quality rather than space allowance. This indicates that EU indoor systems achieve high productivity largely because they minimize environmental variability, a finding that contrasts sharply with African systems, where climate control is limited.

Free-range broiler systems in the EU combine indoor floor housing with access to an outdoor range. EFSA identifies the covered veranda as a crucial design element, serving as a transitional area that encourages birds to use the outdoor range [13]. These systems may involve permanent buildings or mobile units and are typically used in alternative or niche production chains. Outdoor range design strongly influences welfare outcomes. Research shows that vegetation cover, shelter availability, and shade significantly increase range use [19]. EFSA recommends that at least 70% of the range be vegetated, with approximately 50% consisting of trees and shrubs to reduce predation stress and encourage more even use of the area [13]. Comparative analyses also show that outdoor systems may improve behavioural expression but require careful design to avoid welfare trade-offs related to predation, climate exposure, and uneven range use [20]. Fast-growing broilers, which dominate EU production, often show limited use of the range due to reduced mobility and locomotory disorders. As a result, EFSA recommends using slower-growing hybrids in free-range systems to maximize welfare gains [13]. Free-range systems also introduce specific hazards, including predation stress and increased exposure to pathogens and parasites. Appropriate fencing, shelter provision, and range design are therefore essential preventive measures [13]. Synthesis of the reviewed studies shows that free-range systems improve behavioural expression but do not uniformly improve welfare outcomes. Evidence from EFSA [13] and range-use studies [19,20] demonstrates that welfare benefits depend heavily on genotype, vegetation structure, and shelter availability. Fast-growing hybrids consistently underutilize outdoor areas, limiting the potential welfare gains of free-range systems. Thus, the literature indicates that free-range housing is not inherently superior but conditionally beneficial.

Organic chicken production in the European Union is governed by Regulation (EU) 2018/848 on organic production and labeling of organic products, in particular Annex II, Part II (Livestock production rules) [17]. Organic chickens must be born and raised on organic holdings. Where organic animals are not available, non-organic chicks may be introduced under strict conditions and subject to a conversion period, during which organic production rules must be fully applied before products may be marketed as organic (Annex II, Part II, Section 1.3). Housing systems must allow the expression of natural behaviour, including perching, scratching, and dust bathing. Poultry must have permanent access to open-air areas whenever weather and ground conditions permit. Maximum stocking densities are strictly limited. Housing must include littered floors, natural light, and perches, and must avoid multi-tier systems that compromise welfare. Chickens must have access to open-air runs for a significant proportion of their life. Outdoor areas must be mainly covered with vegetation and may not be used for other purposes such as parking or storage. Runs must be sufficiently large to prevent overgrazing and environmental degradation and must include shelters and predator protection. Feed must be 100% organic, with a preference for feed produced on the holding or in the same region. Poultry diets must meet nutritional requirements across different developmental stages and include roughage. Disease prevention in organic chicken production relies primarily on appropriate breed selection, low stocking density, high-quality feed, good hygiene, and biosecurity. Preventive use of chemically synthesized allopathic veterinary medicinal products, including antibiotics, is prohibited. Such products may be used only when necessary to avoid suffering, under veterinary supervision, and with extended withdrawal periods (Annex II, Part II, Section 1.5). Life-cycle assessments indicate that organic and slower-growing systems may offer welfare benefits but often require greater land use and resource inputs, highlighting the need to balance welfare gains with environmental sustainability [21]. Across organic production studies, a recurring theme is the trade-off between enhanced behavioural opportunities and increased exposure to environmental hazards. Organic systems consistently report improved locomotion and activity [17], but also higher risks of parasitism and predation [21]. The literature, therefore, positions organic systems as welfare-enhancing but resource-intensive, requiring careful management to avoid unintended welfare compromises.

#### 3.2.2. Environmental Control in African Systems

**Figure 2:**
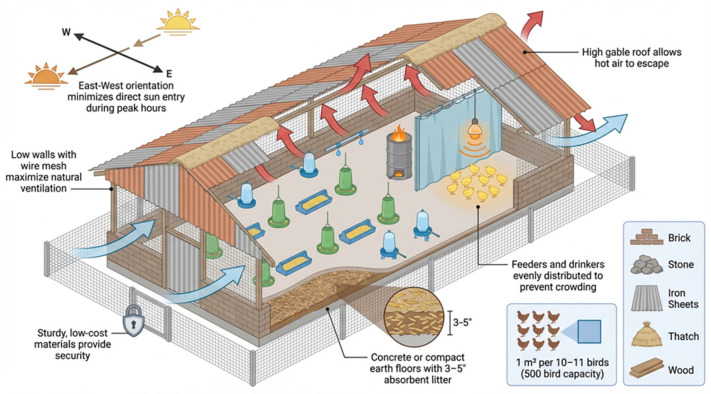
A standard efficient broiler housing system in Sub-Saharan Africa. Source: FigureLabs AI scientific illustration.

It is important to note that broiler production systems across Africa are highly heterogeneous, reflecting substantial variation in climate, infrastructure, market organization, and policy environments across North, West, East, and Southern Africa. Intensive broiler housing in many African countries is primarily found in middle-income and rapidly urbanizing economies such as South Africa, Egypt, Morocco, Nigeria, Kenya, and Ghana. However, the structure and technological sophistication of intensive systems vary substantially across regions. In Southern Africa and parts of North Africa, some commercial farms operate fully enclosed, climate-controlled houses, whereas in West and East Africa, the dominant housing type remains the open-sided or semi-closed barn adapted to natural ventilation and local climatic constraints [22]. Figure 2 presents a representative open-sided broiler house commonly used in Sub-Saharan Africa, highlighting the reliance on natural ventilation and curtain-sided walls. Comparative studies demonstrate the performance limitations of less-controlled housing systems. In Nigeria, broilers reared in deep litter, open-sided systems exhibited significantly lower growth performance compared to those in more controlled cage systems, with housing system effects reaching statistical significance (*p* = 0.029) and higher weekly weight gains (28.0 ± 1.21 g) and final body weights (up to 1.696 kg at 8 weeks) observed under controlled conditions [23]. These findings highlight the strong influence of climate on production outcomes and the challenges of achieving EU-level environmental control in tropical regions. Stocking densities in African intensive systems often approach or exceed EU legal limits, and welfare standards are often not enforced. Heat stress, poor litter quality, and air quality problems are common challenges. While some commercial farms in South Africa and North Africa have adopted fully closed, climate-controlled houses, these remain relatively rare due to high capital and energy costs [22]. A distinct feature of African broiler production is the widespread use of adjustable curtains, which are also a common precision livestock farming technology. These structures improve airflow but also increase exposure to wild birds, pathogens, and weather variability. The physiological vulnerability of broilers to heat stress, due to their limited ability to dissipate heat, makes these systems particularly sensitive to temperature fluctuations [24]. Heat stress is consistently identified as one of the most significant constraints on broiler performance in tropical regions, impairing immunity, reducing feed intake, and compromising carcass quality [25,26,27].

Semi-intensive systems are widespread across West, East, and Southern Africa, but their characteristics differ markedly between regions. In East Africa, for example, semi-intensive units often rely on simple permanent structures built from locally available materials, whereas in West Africa, producers more frequently combine semi-intensive housing with small-scale commercial feed and chick suppliers. Backyard or extensive poultry systems dominate in low-income rural areas across Sub-Saharan Africa, but the degree of confinement, breed types, and management practices vary considerably between countries and even between districts. Thus, while these systems share broad features, they do not represent a uniform production model [22]. These systems typically use simple permanent or semi-permanent structures built from locally available materials such as timber, bricks, corrugated iron sheets, or thatch. Key features include deep-litter housing, manual feeding and watering, natural ventilation, and lower stocking densities than in intensive systems. Performance in semi-intensive systems varies widely and depends heavily on farmer knowledge, feed quality, and disease management. Studies from Tanzania show that smallholder poultry producers face significant constraints related to disease outbreaks, limited access to veterinary services, and inconsistent feed quality [28]. Similar constraints have been documented across East and Southern Africa, where smallholder producers frequently lack access to technical training, quality feed, and structured disease-control programs [29,30]. These challenges are further compounded by the limited availability of agricultural Extension services across much of rural Africa. In many regions, farmers have little or no access to trained Extension officers who could provide practical guidance on housing improvements, flock health management, and basic biosecurity. The absence of such support systems contributes to persistent knowledge gaps, reduces the adoption of improved practices, and limits the overall productivity and resilience of semi-intensive and smallholder broiler operations. Backyard or extensive poultry systems dominate in low-income rural areas and are often classified as village or traditional systems. Although these systems primarily house indigenous chickens, small numbers of broilers are increasingly incorporated for household consumption and local markets. Housing typically consists of simple night shelters made from mud, wood, or woven materials. Birds may be confined at night and allowed to scavenge during the day. Indigenous breeds used in these systems are more resilient to heat, disease, and predation than commercial hybrids [31], but broilers perform poorly under scavenging conditions due to their high nutrient requirements and limited mobility. Backyard systems have very low input costs but face high mortality rates due to disease, predation, and extreme weather. Despite these challenges, they play an important role in rural livelihoods, food security, and women’s empowerment [32]. Village poultry systems remain central to rural livelihoods across sub-Saharan Africa, providing income, nutrition, and social capital despite high mortality rates and limited access to veterinary care [9,33].

Across African intensive systems, the reviewed studies converge on a central finding: heat stress is the dominant constraint shaping welfare and productivity. Experimental evidence from Nigeria [23] and broader regional analyses [22,25,26,27] consistently show that open-sided housing systems, which rely on natural ventilation, are unable to maintain thermal comfort during peak heat periods. This results in reduced growth performance, increased mortality, and compromised welfare outcomes. In contrast, as summarized in Table 1, European Union (EU) systems are characterized by fully enclosed, climate-controlled housing equipped with mechanical ventilation, heating, and cooling systems that maintain relatively stable indoor conditions. This environmental stability mitigates the effects of external climatic variability and supports more consistent welfare and productivity outcomes. The comparison highlights a fundamental divergence between regions: African systems prioritize climate adaptation through low-cost, naturally ventilated designs, whereas EU systems rely on technological control to stabilize environmental conditions.

### 3.3. RQ2. How Do These Environmental Control Strategies Influence Key Welfare and Production Outcomes Under Temperate Versus Tropical Climatic Conditions?

#### Effect of Heat Stress on Broiler Chicken Production

Broilers are physiologically ill-equipped to cope with high temperatures due to their feather coverage, lack of sweat glands, and high metabolic heat production. Heat stress impairs immunity, reduces feed intake, and increases mortality [24]. These physiological limitations make climate control a critical component of housing design. In the EU, mechanical ventilation, evaporative cooling, and automated climate control systems maintain stable indoor conditions [12]. In contrast, African systems often rely on natural ventilation due to high energy costs, unreliable electricity supply, and limited access to cooling technologies [22]. Environmental management strategies in Africa include orienting houses to minimize solar gain, using reflective roofing materials, increasing air exchange through open-sided designs, strategically placing shade trees, and frequently turning litter to reduce moisture buildup. However, these strategies cannot fully mitigate the impacts of extreme heat events, which are becoming more frequent due to climate change. As a result, heat stress remains one of the most significant constraints on broiler production in Africa. Synthesis of the evidence indicates that semi-intensive systems exhibit the greatest variability in outcomes. Studies from Tanzania [28] and other regions [29,30] show that performance is strongly dependent on farmer knowledge, feed quality, and disease management capacity. This variability reflects structural constraints, like limited veterinary access, inconsistent feed supply, and weak extension services, which are repeatedly documented across the literature.

### 3.4. Socio-Economic Drivers of Broiler Production in Africa and the EU

**Figure 3:**
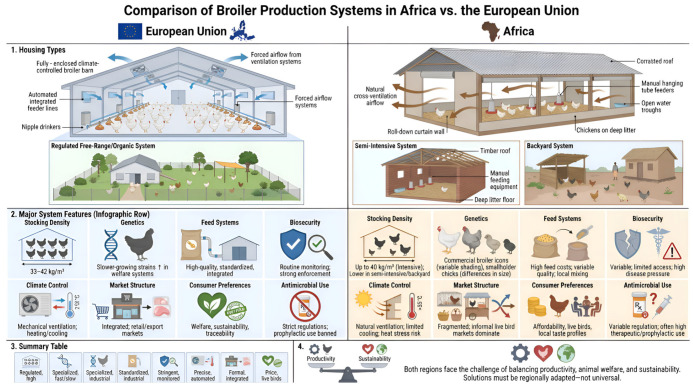
Comparison of broiler production systems. Source: FigureLabs AI scientific illustration.

A visual comparison of dominant broiler production systems in Africa and the EU is provided in Figure 3, illustrating key structural and environmental differences. In Sub-Saharan Africa, smallholder poultry production plays a crucial role in rural livelihoods, although the scale, market integration, and access to inputs vary substantially across West, East, and Southern Africa. For example, West African producers often face high feed prices and fragmented markets, whereas Southern African producers operate within more structured commercial supply chains. These intra-regional differences mean that “African broiler production” encompasses a wide spectrum of systems rather than a single continental model [32]. These systems are characterized by low input costs but also face significant challenges, including high feed prices, limited access to credit, poor veterinary infrastructure, fragmented markets, and high disease pressure [28]. Value-chain analyses highlight that fragmented markets, weak governance structures, and inconsistent service delivery further constrain the development of African poultry sectors [34,35]. In contrast, the EU poultry sector is highly integrated, with strong coordination across breeding, feed production, processing, and retail [36]. This integration enhances efficiency, reduces production costs, and supports compliance with strict welfare and environmental regulations. EU producers also benefit from stable markets and strong consumer demand for welfare-oriented products. These socio-economic differences shape the evolution of housing systems in each region. While the EU can invest in high-tech climate-controlled barns, African producers often prioritize low-cost, climate-adaptive systems that align with local economic realities. Biosecurity limitations are particularly acute in low-resource settings, where farmers often lack access to veterinary services, diagnostic tools, and structured disease-prevention programs [37,38]. Across all reviewed studies, socio-economic constraints emerge as a cross-cutting determinant of system performance in Africa. High feed costs, fragmented markets, and limited access to credit [28,34,35] consistently restrict producers’ ability to invest in improved housing or biosecurity. In contrast, EU studies highlight the stabilizing role of vertically integrated supply chains [36], which reduce variability and support compliance with welfare regulations. This divergence in structural conditions underpins many of the differences observed between regions.

## 4. Discussion

### 4.1. RQ1. How Do Environmental Control Strategies Differ Between Broiler Housing Systems in Africa and the EU?

#### 4.1.1. Broiler Production Comparison Across Regions (Africa & EU)

Broiler production in Africa involves a mix of modern intensive systems and traditional backyard farming [1,9]. However, production structures differ substantially across North, West, East, and Southern Africa, reflecting variation in climate, infrastructure, and market integration. Industry fragmentation, limited resource availability, and varying financial opportunities widen disparities in production scale and efficiency. Smallholder farmers play an essential role, yet they face challenges, including access to quality feed, veterinary services, and disease management [1]. In contrast, the EU production structure is highly organized and vertically integrated, supported by advanced technology, precision management, and strict regulatory oversight [7]. These regulations influence housing construction, management practices, and breeding strategies. EU consumers increasingly demand higher welfare standards, driving adoption of enriched systems, slower-growing strains, and organic production [8]. African consumers, however, prioritize affordability, resulting in the dominance of conventionally raised broilers [1]. Yet, as incomes rise, an emerging middle class shows a growing interest in higher-quality products and local taste profiles [39,40,41]. Both regions face distinct challenges. In Africa, the informal nature of poultry farming complicates disease control and biosecurity, while high feed costs and limited technical expertise constrain growth [28]. In the EU, intensive production raises concerns about antimicrobial resistance, environmental impacts, and welfare issues associated with fast-growing strains [7]. However, the EU’s strong research infrastructure supports innovation in welfare monitoring, environmental enrichment, and sustainable production [14,17]. Recent EFSA evaluations emphasize hazard-based welfare assessment and recommend substantially lower stocking densities than current legal thresholds, reflecting updated scientific understanding of welfare risks [13].

EU indoor production is dominated by fully enclosed, climate-controlled barns with strict regulatory thresholds for stocking density, air quality, and lighting [11,13]. Despite this high level of control, welfare challenges persist due to the biological limitations of fast-growing strains. African indoor systems are far more heterogeneous. While some commercial farms use enclosed barns, the dominant model is the open-sided house, which relies on natural ventilation and climate-adaptive design [22]. While these systems mitigate heat stress, they offer limited control over humidity, ammonia, and pathogen exposure. Comparative studies show that broilers in open-sided houses perform worse than those in controlled environments [23]. Outdoor access systems in the EU, including free-range and organic production, are tightly regulated under EU law [42]. Range design, vegetation, and shelter strongly influence welfare and range use [19]. Slower-growing strains are recommended to reduce locomotory problems [13]. In Africa, outdoor access is common in semi-intensive and backyard systems. These systems offer behavioral freedom but are poorly suited to fast-growing broilers, which struggle with mobility, heat stress, and predation [31]. Indigenous breeds perform better in these systems due to their resilience. While broiler production systems in the EU are often considered benchmarks for efficiency and animal welfare, their direct transfer to African contexts is constrained by significant biophysical, economic, and institutional differences. As such, the adoption of EU-style housing systems in Africa requires a critical evaluation of trade-offs, scalability, and long-term feasibility.

#### 4.1.2. Comparative Synthesis

Across the reviewed literature, a clear divergence emerges between EU and African environmental control strategies. EU systems rely on fully enclosed, mechanically ventilated barns that maintain stable temperature, humidity, and air quality, enabling consistent welfare outcomes even for fast-growing genotypes. In contrast, African systems depend heavily on natural ventilation through open-sided houses, which exposes birds to heat stress, humidity fluctuations, and pathogen pressure. These differences are not merely technological but structural: EU systems operate within integrated supply chains and strict regulatory frameworks, whereas African systems must adapt to climatic extremes, limited capital, and inconsistent energy access. Collectively, the evidence shows that environmental control is the primary axis along which regional production systems diverge, shaping both welfare and productivity. Moreover, the feasibility of environmental control in African systems is constrained not only by climate but also by infrastructural limitations, including unreliable electricity supply, high energy costs, and limited access to mechanical ventilation technologies. These constraints differ markedly between regions, with Southern Africa showing greater adoption of controlled-environment housing, while West and East Africa rely predominantly on natural ventilation. These regional differences underscore the need to evaluate not only how African systems differ from EU systems, but also under which specific conditions elements of EU-style environmental control can be feasibly adopted, partially adapted, or remain impractical in African contexts.

### 4.2. RQ2. How Do These Environmental Control Strategies Influence Key Welfare and Production Outcomes Under Temperate Versus Tropical Climatic Conditions?

#### 4.2.1. Economic vs. Welfare Trade-Offs

**Figure 4:**
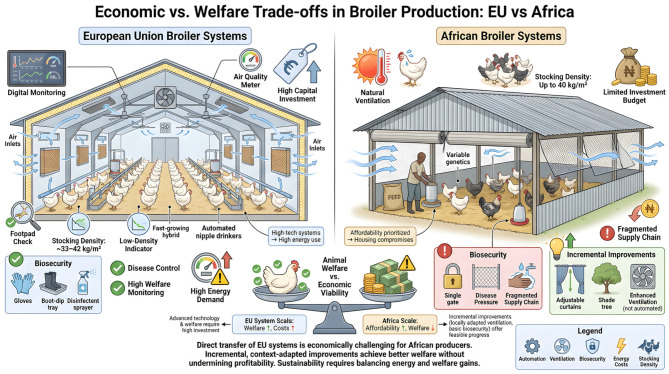
Economic vs. Welfare Trade-offs in broiler production: EU vs. Africa. Source: FigureLabs AI scientific illustration.

A central challenge in adapting EU production models lies in balancing improvements in animal welfare with economic viability. EU systems are typically capital-intensive, reflecting high fixed investments in climate-controlled housing and automation (Figure 4). Life-cycle and economic analyses indicate substantially higher capital and energy inputs per unit of output compared to less-controlled systems, although these are partially offset by improved feed conversion and lower mortality rates [21,43,44]. These features enhance welfare outcomes, such as reduced stocking density stress and improved disease control, but require substantial upfront investment and ongoing operational costs. In contrast, African systems operate under significant financial and structural constraints, including high feed costs and limited access to credit, which have been identified as primary bottlenecks to productivity in multiple value-chain analyses [34,35]. Under these conditions, prioritizing affordability often results in compromises in housing quality and environmental control. Consequently, a direct transition to EU-standard systems may not be economically sustainable for small- and medium-scale producers. Instead, incremental improvements, such as enhanced ventilation, locally adapted housing designs, and basic biosecurity measures, may offer more feasible pathways for improving welfare without undermining profitability. Environmental impact assessments further indicate that high-tech systems may carry greater energy demands unless supported by efficient technologies, highlighting the need to balance welfare improvements with sustainability considerations [43,44].

Across both regions, the trade-off between welfare and economic feasibility is mediated by structural conditions: EU producers operate within integrated supply chains that can absorb high capital costs, whereas African producers face fragmented markets and limited financing. This structural divergence explains why welfare-enhancing technologies are widely adopted in the EU but only selectively feasible in African contexts. The scalability of EU production technologies in African settings is further limited by infrastructure gaps and contextual variability. Technologies such as climate-controlled housing, automated feeding systems, and precision livestock monitoring depend on reliable electricity, technical expertise, and stable input supply chains, conditions that remain inconsistent across many regions of Africa [22,35]. Moreover, the diversity of production systems, from smallholder farms to emerging commercial farms, requires flexible, context-specific solutions rather than standardized technological packages. As a result, technology transfer should be conceptualized as adaptation rather than replication. Hybrid systems integrating selected elements of EU practices with locally appropriate innovations, such as improved natural ventilation or low-cost environmental modifications, have been identified as more feasible pathways for incremental gains in productivity and welfare under resource-constrained conditions [22,35]. These findings indicate that the feasibility of adopting EU-style systems is not uniform across Africa but depends on a constellation of enabling conditions, including energy reliability, capital availability, and technical capacity. This reinforces the need for a structured framework identifying when EU technologies are feasible, infeasible, or only partially adaptable.

#### 4.2.2. Climate Change Implications

**Figure 5:**
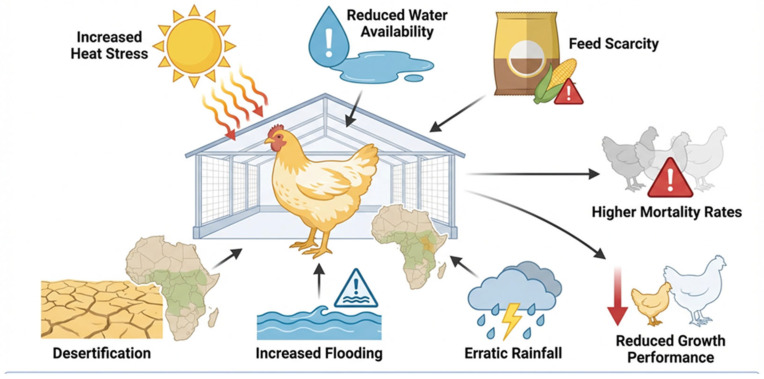
Climate Change Implications on Broiler Production in Sub-Saharan Africa. Source: Authors’ illustration generated using the FigureLabs AI scientific illustration platform.

Climate change adds an additional layer of complexity, particularly for African production systems already vulnerable to heat stress and environmental variability. Rising temperatures and increased frequency of extreme weather events are likely to exacerbate mortality rates, reduce feed efficiency, and intensify disease pressures in broiler production. While EU systems are better equipped to buffer climatic fluctuations through controlled environments, their high energy demands raise concerns regarding sustainability and carbon footprint. In African contexts, where energy access is limited and costly, reliance on such systems may not be viable at scale. Instead, climate-adaptive housing and management strategies will become increasingly important (Figure 5). Projected increases in heat-load indices across sub-Saharan Africa are expected to intensify these challenges, making climate-adaptive housing and management strategies essential for future resilience [24]. These climate-related constraints further reduce the feasibility of transferring EU-style systems to many African regions, reinforcing the need for region-specific adaptation rather than replication.

#### 4.2.3. Role of Extension Services in Strengthening Broiler Production in Africa

A major structural limitation affecting broiler production across much of Africa is the limited availability of agricultural extension services, particularly in rural areas where technical support networks are weakest. Studies on smallholder poultry systems consistently highlight gaps in access to veterinary services, training, and advisory support as key constraints on productivity and disease control [28,29,30]. The absence of effective extension systems contributes to suboptimal housing design, poor biosecurity practices, and delayed responses to disease outbreaks, thereby increasing mortality and reducing production efficiency [35,37]. In low-resource settings, where farmers often rely on informal knowledge networks, the lack of structured technical guidance further limits the adoption of improved practices. Strengthening extension capacity, through trained personnel, mobile advisory systems, and community-based approaches, has been identified as a cost-effective strategy for improving poultry health, productivity, and animal welfare in smallholder systems [35,38,45]. Such interventions are particularly important for facilitating the uptake of incremental innovations tailored to local production conditions. Extension services, therefore, play a pivotal role in determining whether hybrid or partially adapted EU-style systems can be successfully implemented in African contexts.

#### 4.2.4. Comparative Synthesis

The reviewed studies consistently demonstrate that climatic context amplifies or mitigates welfare risks. In temperate EU conditions, controlled ventilation, regulated lighting, and strict air-quality thresholds reduce the incidence of footpad dermatitis, hock burn, and gait abnormalities. Conversely, in tropical African climates, heat stress is the dominant constraint on welfare and production, reducing feed intake, impairing immunity, and increasing mortality. Semi-intensive and backyard systems show even greater variability due to inconsistent feed quality, limited veterinary access, and management constraints. Overall, the evidence indicates that welfare outcomes are strongly mediated by the interaction between climate and environmental control capacity: where climate control is robust, welfare risks are minimized; where it is limited, welfare and productivity decline sharply. This comparative evidence underscores that environmental control capacity, not simply housing type, determines welfare outcomes across regions. This finding provides the conceptual foundation for identifying the conditions under which EU-style systems can be feasibly transferred to African contexts.

#### 4.2.5. Conditions for Feasible, Infeasible, and Partial Transfer of EU Systems

The feasibility of transferring EU-style broiler housing systems to African contexts depends on a combination of climatic, infrastructural, economic, and institutional conditions. The reviewed evidence indicates that transferability is not binary but falls along a continuum from fully feasible to entirely impractical, with a large intermediate space where partial adaptation is possible. EU-style systems are feasible where electricity supply is reliable and affordable, technical expertise is available, supply chains are integrated, cooling technologies can operate effectively, and capital investment is accessible. Typically, those elements can be implemented in parts of Southern and North Africa. Full replication is infeasible where heat and humidity exceed cooling capacity, electricity is unreliable, energy costs are prohibitive, veterinary infrastructure is weak, and farm-gate margins are low. These conditions are common in the West, the East, and rural Sub-Saharan Africa. Hybrid systems are viable where open-sided houses can be upgraded, low-cost cooling can be used intermittently, natural ventilation can be optimized, and heat-tolerant or slower-growing strains are used.

## 5. Strengths and Limitations of the Study

This study has several strengths. First, it provides a structured, multidimensional comparison of broiler production systems across two contrasting regions, addressing a gap in the literature, where analyses are often limited to single regions or isolated production aspects. Second, the use of a consistent analytical framework enables meaningful comparison across housing systems, environmental management, welfare outcomes, and socio-economic drivers. Third, integrating the recent literature enhances the relevance of the findings. A further strength of this review is the incorporation of an explicit analytical framework identifying the conditions under which EU-style systems are feasible, infeasible, or only partially adaptable in African contexts. This responds to a key gap in previous comparative studies, which often describe regional differences without specifying the structural factors that determine system transferability. However, several limitations should be acknowledged. The study is based on a narrative review of the existing literature, which may introduce bias related to study selection and data availability. In particular, the heterogeneity of African production systems and the limited availability of standardized data constrain direct comparisons. These limitations are especially pronounced given the substantial variation across North, West, East, and Southern Africa, where climatic, infrastructural, and market conditions differ markedly. Additionally, differences in methodologies and indicators across studies may affect the consistency of the analysis. Future research should focus on generating standardized, empirical data across regions and developing integrated assessment frameworks that combine productivity, welfare, environmental, and economic indicators. Such frameworks would also support the development of operational definitions of context-specific systems, enabling clearer guidance on how housing and management strategies should be adapted to regional constraints rather than relying on generalized recommendations.

## 6. Conclusions

Broiler production systems in Africa and the European Union differ substantially in their structural design, environmental control capacity, welfare outcomes, and regulatory oversight. These differences reflect broader climatic, economic, and institutional contexts that shape which housing and management strategies are feasible in each region. While EU systems benefit from advanced climate control, strong regulatory enforcement, and integrated supply chains, African systems operate across a wide spectrum—from fully enclosed commercial houses in parts of Southern and North Africa to semi-intensive and backyard systems in West and East Africa. Despite these contrasts, both regions face shared challenges in balancing productivity, animal welfare, and sustainability. The comparative evidence presented in this review demonstrates that welfare outcomes are primarily determined by environmental control capacity rather than housing type alone. This finding underscores the importance of evaluating not only how systems differ, but why these differences emerge and under which conditions specific technologies or management practices can be transferred across regions. Overall, the findings of this review highlight the need for adaptive, region-specific strategies rather than universal housing models. Future research should focus on generating standardized empirical data across regions and developing integrated assessment frameworks that combine welfare, productivity, environmental sustainability, and economic feasibility. Such efforts will support the development of resilient broiler production systems capable of meeting growing demand under diverse and changing climatic conditions.

## Figures and Tables

**Table 1:** Comparison of Broiler System Characteristics: EU vs. Africa.

System Feature	European Union	Africa
Housing Type	Fully enclosed, climate-controlled barns; regulated free-range and organic housing systems.	Open-sided or semi-closed houses; a mix of intensive, semi-intensive, and backyard housing systems.
Stocking Density	33–42 kg/m^2^ depending on compliance.	Up to 40 kg/m^2^ in intensive systems; lower in semi-intensive/backyard.
Genetics	Fast-growing commercial hybrids; increasing use of slower-growing strains in welfare-focused systems.	Commercial hybrids in intensive farms; variable quality in smallholder systems.
Feed Systems	High-quality, standardized feed; integrated supply chains.	High feed costs; variable quality; local mixing common in smallholder systems.
Biosecurity	Strong enforcement, routine monitoring, and outcome-based welfare indicators.	Highly variable; limited veterinary access; high disease pressure.
Climate control	Mechanical ventilation, heating, cooling; stable indoor environment.	Natural ventilation; limited cooling; high heat stress risk.
Market Structure	Highly integrated; strong retail and export markets.	Fragmented; dominance of informal live bird markets.
Consumer preferences	Welfare, sustainability, traceability.	Affordability, live birds, and local taste profiles.

**Table 2:** (**a**). Summary of Constraints. (**b**). Implications for Production.

(a)		
Constraint Domain	European Union (EU)	Africa
Biophysical	Temperate climate; controlled indoor environments; low pathogen exposure	Hot/humid climates; open-sided housing; high pathogen pressure; heat stress risk
Economic	Integrated supply chains; stable feed markets; high capital access; automation	Fragmented markets; high feed costs; limited credit; smallholder dominance
Institutional	Strong regulatory enforcement, developed veterinary systems, and welfare monitoring	Weak enforcement; limited veterinary infrastructure; informal market systems
Socio-cultural	Demand for welfare, sustainability, and traceability	Price-driven demand; preference for live birds; emerging quality segment
**(b)**		
**Constraint Domain**	**Key Implications for Broiler Production**	
Biophysical	Environmental control is optimized in the EU, while African systems are constrained by climate and limited cooling capacity	
Economic	High capital requirements limit the adoption of advanced housing technologies in Africa	
Institutional	Weak enforcement reduces consistency in welfare standards and disease control in African systems	
Socio-cultural	Market incentives in the EU support welfare improvements, whereas African systems prioritize affordability	

## Data Availability

No new data were created or analyzed in this study. Data sharing is not applicable to this article.
